# Direct quantitative material decomposition employing grating-based X-ray phase-contrast CT

**DOI:** 10.1038/s41598-018-34809-6

**Published:** 2018-11-06

**Authors:** Eva Braig, Jessica Böhm, Martin Dierolf, Christoph Jud, Benedikt Günther, Korbinian Mechlem, Sebastian Allner, Thorsten Sellerer, Klaus Achterhold, Bernhard Gleich, Peter Noël, Daniela Pfeiffer, Ernst Rummeny, Julia Herzen, Franz Pfeiffer

**Affiliations:** 1 Chair of Biomedical Physics, Department of Physics and Munich School of BioEngineering, Technical University of Munich, 85748 Garching, Germany; 2 Department of Diagnostic and Interventional Radiology, Klinikum rechts der Isar, Technical University of Munich, 81675 München, Germany; 3 Max-Planck-Institute of Quantum Optics, Hans-Kopfermann-Straße 1, 85748 Garching, Germany

**Keywords:** Preclinical research, Biomedical engineering

## Abstract

Dual-energy CT has opened up a new level of quantitative X-ray imaging for many diagnostic applications. The energy dependence of the X-ray attenuation is the key to quantitative material decomposition of the volume under investigation. This material decomposition allows the calculation of virtual native images in contrast enhanced angiography, virtual monoenergetic images for beam-hardening artifact reduction and quantitative material maps, among others. These visualizations have been proven beneficial for various diagnostic questions. Here, we demonstrate a new method of ‘virtual dual-energy CT’ employing grating-based phase-contrast for quantitative material decomposition. Analogue to the measurement at two different energies, the applied phase-contrast measurement approach yields dual information in form of a phase-shift and an attenuation image. Based on these two image channels, all known dual-energy applications can be demonstrated with our technique. While still in a preclinical state, the method features the important advantages of direct access to the electron density via the phase image, simultaneous availability of the conventional attenuation image at the full energy spectrum and therefore inherently registered image channels. The transfer of this signal extraction approach to phase-contrast data multiplies the diagnostic information gained within a single CT acquisition. The method is demonstrated with a phantom consisting of exemplary solid and fluid materials as well as a chicken heart with an iodine filled tube simulating a vessel. For this first demonstration all measurements have been conducted at a compact laser-undulator synchrotron X-ray source with a tunable X-ray energy and a narrow spectral bandwidth, to validate the quantitativeness of the processing approach.

## Introduction

The idea to use the energy dependence of X-ray attenuation for the differentiation of materials with similar Hounsfield units (HU) dates back to the end of the 1970s^1^. Although, technical difficulties like resolution, movement artifacts and instabilities prevented the immediate clinical implementation^2,3^, the diagnostic potential has already been predicted in early studies on electron density and effective atomic number of lesions, cysts or tissue degradations in comparison to healthy tissue^4–6^. The unambiguous differentiation of two specific materials is a common issue in medical imaging. Calcium, contrast agent, coagulated blood or kidney stones are just a few examples for materials with similar HUs but different atomic numbers or electron densities. During the last decade, the dual-energy technology has successfully been implemented into commercial CT systems and many studies show the benefit for related clinical challenges in stroke diagnosis, pulmonary perfusion imaging or bone mineral density determination, among others^7–10^. Depending on the technical realization, these machines use dual-source or dual-(detector-) layer techniques or rapid kVp-switching^11^. With the latest progress in X-ray detector fabrication, the current research focus lies on the implementation of multi-threshold photon counting detectors to realize spectral CT^12^. Independently of the image acquisition method, the techniques utilize the dual information to transform the image data into different frames of reference. This opens up a whole range of visualization methods optimized for specific diagnostic tasks, namely virtual monoenergetic images, effective atomic number maps, quantitative iodine content or virtual non-contrast images.

Parallel to this development, a novel X-ray imaging technique has been established in preclinical research - grating-based X-ray phase-contrast. Additionally to the conventional attenuation image, it provides access to the phase shift and an additional small-angle scattering signal, by using a three-grating interferometer also at conventional X-ray tubes as implemented in clinical X-ray scanners^13–15^. Thereby, the phase signal is proportional to the electron density and in combination with the attenuation coefficient from the conventional image comprises the same duality as achieved by dual-energy measurements but in a more direct way. The simultaneous acquisition ensuring the inherent image registration as well as the direct access to the electron density indicate potential advantages of this approach. It has been shown previously, that quantitative phase-contrast CT allows the determination of the effective atomic number of materials by relating it to the ratio of the total attenuation coefficient and the measured phase shift either by fitting a power function^16^ or by interpolation of the tabulated interaction cross sections for the relevant atomic numbers^17^. Here we use a simple model for the parametrization of the underlying photon interactions which was originally proposed by *Alvarez et al*.^1^ to calculate the effective atomic numbers and virtual monoenergetic images. Subsequently, we change the basis system mathematically to a system spanned by two different basis materials for the purpose of quantitative material decomposition. Such material decompositions are currently used to discriminate specific materials in clinical imaging^7–10^. The aim of this study is to demonstrate that the duality of the phase and attenuation images allows to obtain the same diagnostic information as the dual-energy technique and to highlight potential advantages of this approach.

## Results

### Attenuation and phase-contrast information

**Figure 1:**
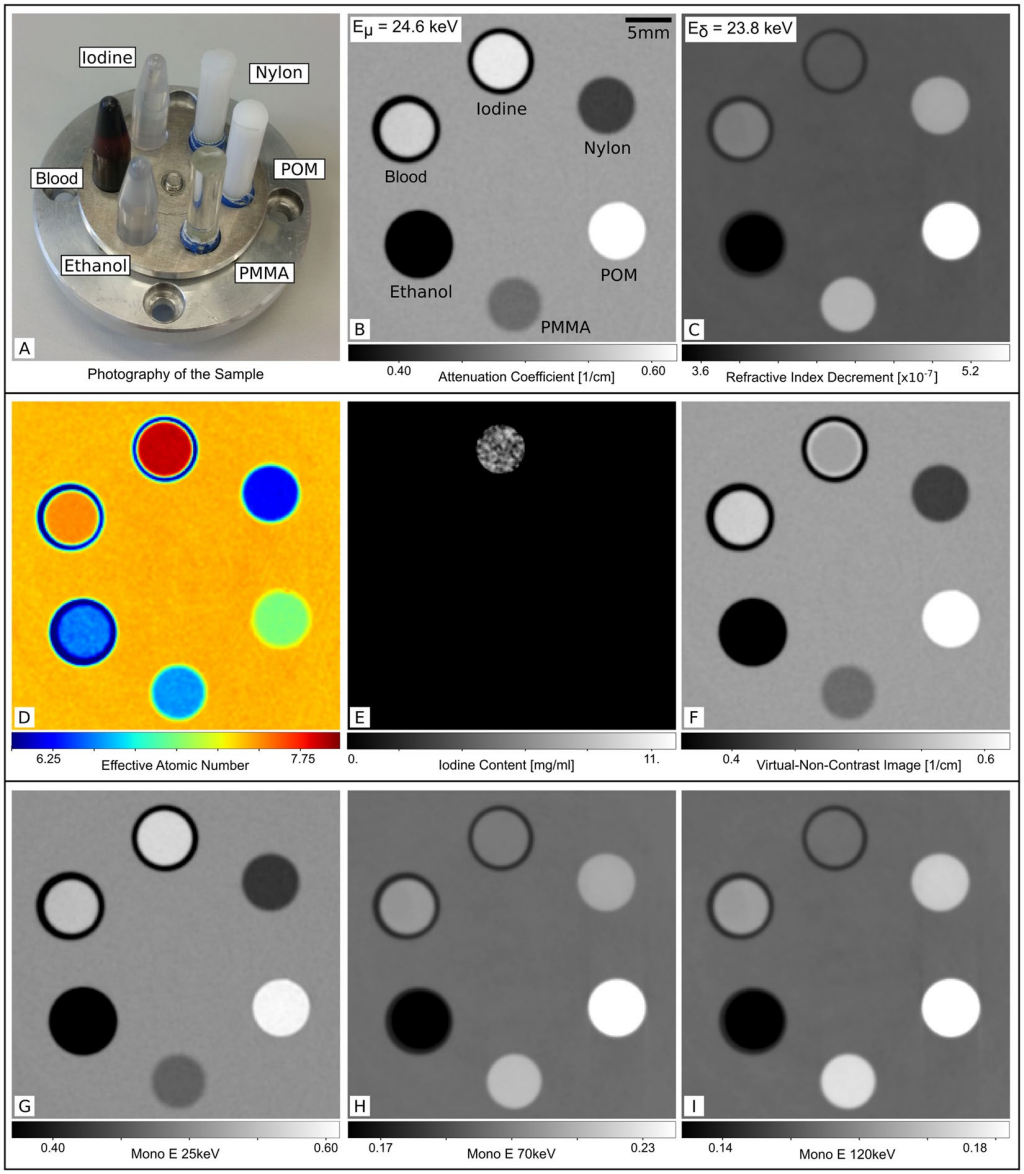
Measurements and results of virtual dual-energy processing for a material phantom. (**A**) Photography of the sample consisting of 6 different solid or liquid materials. From the reconstructed attenuation (**B**) and phase-contrast (**C**) data an effective interaction energy of ${E}_{{\rm{eff}}}^{{\rm{\mu }},{\rm{\delta }}}=\mathrm{24.6,}\,23.8\,{\rm{keV}}$ was assigned via the literature value for the linear attenuation coefficient and the refractive index decrement of PMMA. The effective atomic number map (**D**) shows the distribution of *Z*_eff_ ≈ 6.25 for Nylon to *Z*_eff_ ≈ 7.99 for iodine. The quantitative iodine map (**E**) shows positive values of [I] ≈ 4.6 mg/ml only for the iodine solution. The virtual non-contrast image (**F**) is the conventional attenuation image where the identified iodine containing pixels are replaced with the attenuation value of water. The virtual monoenergetic image at ${E}_{1}^{{\rm{VMI}}}=25\,{\rm{keV}}$ (**G**) looks very similar to the conventional attenuation image. For the higher energies (**H**,**I**), the contribution of the electron density increases and the virtual monoenergetic image at ${E}_{{\rm{3}}}^{{\rm{VMI}}}=120\,{\rm{keV}}$ looks very similar to the electron density image (which is simply proportional to the refractive index decrement image).

The image results of the measurement of the material phantom are presented in Fig. 1. Via comparison of the measured linear attenuation coefficient (*μ*) and refractive index decrement (*δ*) of a calibration PMMA cylinder with the tabulated literature values from the NIST data base^18^ (see Table 1), an effective energy has been assigned to the whole measurement. The energy calibration yields an effective energy of *E*_eff_ = 24.6 ± 0.2 keV for the attenuation image and *E*_eff_ = 23.8 ± 0.2 keV for the phase-contrast image. The different results for the calibration energies are due to the different underlying interaction mechanisms but still in the expected range for a measured mean energy of *E*_eff_ = 24.3 keV at the position of the sample with the given energy bandwidth of about 3% of the compact light source. In the conventional attenuation image, ethanol, PMMA and nylon appear darker than water - blood, POM and the iodine solution appear brighter. This is in accordance with the different linear attenuation coefficients of these materials. In the refractive index decrement image ethanol appears darker than water, PMMA, POM, nylon and blood appear brighter and iodine has the same gray level as water in accordance with the different phase shifting properties of the respective materials. This demonstrates the complementarity of phase-contrast and conventional attenuation imaging.

**Table 1:** Comparison of measured linear attenuation coefficients *μ*, and refractive index decrements *δ* with literature values for the material phantom at an effective energy of E_eff_ = 24.6 keV for the conventional attenuation image and E_eff_ = 23.8 keV for the phase-contrast image.

	*μ*_m_ [cm^−1^]	*μ*_1_ [cm^−1^]	*δ*_m_ [10^−7^]	*δ*_1_ [10^−7^]
NaI (5.9) mg/ml	0.597 ± 0.006	0.609	4.08 ± 0.07	4.07
Blood	0.568 ± 0.006	0.574	4.31 ± 0.07	4.27
Ethanol	0.323 ± 0.006	0.325	3.23 ± 0.06	3.27
PMMA	0.470 ± 0.006	0.470	4.70 ± 0.06	4.70
POM	0.628 ± 0.006	0.628	5.55 ± 0.06	5.55
Nylon	0.423 ± 0.006	0.419	4.61 ± 0.06	4.58
Water	0.523 ± 0.006	0.523	4.07 ± 0.06	4.07

The subscripts m and l indicate measured and literature values. The literature values are calculated from tabulated values from the NIST database^25^, from^26^ for blood and from^20^ for the NaI solution. The given uncertainty includes the standard deviation of the image region and the systematic error.

The measured values of linear attenuation coefficient and refractive index decrement have been compared to theoretical values^18–20^ (Table 1). The measured values in regions of interest of 20 × 20 pixel for the well-defined materials (i.e. all materials but blood and the iodine solution) show a high quantitative accuracy of less than 0.9% deviation from the literature for *μ* and less than 0.6% for *δ*. Systematic errors on *μ* and *δ* originating from inaccuracies of the interferometer geometry have been evaluated with Gaussian error analysis and were found to be smaller than the standard deviation of the respective measured values. The given error margins in Table 1 include the systematic error resulting from the inaccuracy of the effective energy determination and the statistical error in the respective image region. The literature values for blood refer to physiological uncoagulated blood and therefore show slightly larger differences from the measured values, which were obtained for coagulated blood. The deviations of the values for iodine are attributed to an inaccuracy of the NaI concentration. In an in-depth analysis of the NaI-solution preparation accuracy, it has been seen, that the measured concentrations are systematically 7–14% lower than the nominally prepared concentrations. This could be caused by the hygroscopic behavior of sodium iodide which can lead to an overestimation of the absolute NaI weight during the preparation. The measured attenuation value of the shown NaI solution corresponds to an actual concentration of [NaI] ≈ 5.1 mg/ml (nominally prepared concentration was [NaI] = 5.9 mg/ml).

### Transformation to *Z*_eff_ and *ρ*_e_

With the parametrization of the photon interactions described in the materials and methods section, an effective atomic number can be calculated from the reconstructed *μ* and *ρ*_e_ data sets for every image point. According to the common presentation for clinical dual-energy CT, the effective atomic number is displayed in a pseudo color map (Fig. 1D). At the boarders of the tubes, the effective atomic number values show an overshoot like artifact. This can be related to partial volume effects occurring in regions of the original reconstruction of *μ* and *δ* where a strong contrast is present within one pixel or - due to imperfect alignment - several pixels. As these values are quantitatively not correct they do not follow the relation given by equation 5. Thus, the effective atomic number can not be displayed correctly at the image voxels concerned by partial volume effects resulting in a visual edge enhancement. Table 2 compares the averaged effective atomic numbers for the seven measured materials with literature values. For compounds and mixtures, the effective atomic number is only an auxiliary quantity and there are slightly different definitions for its calculation. Thus, two literature values are given for each material, if available. The measured values are systematically larger (<2% deviation) than the values from both literature sources. For a detailed comparison of the different methods for effective number determination we refer to^21^. The phase signal provides direct access to the electron density via Eq. 1 and thus direct access to this energy independent variable which can be used for further image transformations and material decomposition.

**Table 2:** Quantitative results for the effective atomic numbers **Z**_eff_ and the electron density *ρ*_e_ for the material phantom in comparison to different literature values.

	*Z*_eff,m_	${{\boldsymbol{Z}}}_{{\bf{eff}}{\boldsymbol{,}}{{\bf{l}}}_{{\bf{1}}}}$	${{\boldsymbol{Z}}}_{{\bf{eff}}{\boldsymbol{,}}{{\bf{l}}}_{{\bf{2}}}}$	*ρ*_e,m_⋅[10^29^ m^−3^]	*ρ*_e,l_
NaI (5.9 mg/ml)	7.97 ± 0.06	—	—	3.34 ± 0.07	—
Blood	7.60 ± 0.06	—	7.74	3.54 ± 0.08	—
Ethanol	6.52 ± 0.07	6.35	—	2.69 ± 0.07	2.68
PMMA	6.58 ± 0.03	6.47	6.56	3.86 ± 0.06	3.86
POM	7.05 ± 0.05	6.95	7.03	4.56 ± 0.06	4.56
Nylon	6.24 ± 0.06	6.12	6.21	3.79 ± 0.06	3.76
Water	7.51 ± 0.05	7.42	7.51	3.34 ± 0.06	3.34

The subscript m indicates measured values. For the effective atomic number two different literature sources are given, denoted with l_1_ for values from Qi *et al*.^16^ and l_2_ for values from the XmuDat library^25,26^. The given uncertainty includes the standard deviation of the image region and the systematic error.

### Virtual non-contrast image

Starting from an image base which is mathematically spanned by the effective atomic number and the electron density, a vector basis transformation has been performed. Such a transformation enables the visualization of the image in means of a new basis, e.g. the contribution of two different materials to the image formation. For Fig. 1E the iodine and the blood content have been chosen as basis materials to address the specific clinical case of iodine and blood separation. By that, a quantitative iodine map can be obtained which provides the iodine content in mg/ml. The vector transformation is based on the assumption that every material in the phantom can be represented as a combination of iodine and water. Therefore it leads to negative values in the iodine map for most of the phantom materials. Via this iodine map, the image regions with positive iodine content can be identified and are replaced by the attenuation values of water in the conventional attenuation image. This leads to a very simple so called virtual non-contrast (VNC) image which is a specific feature of dual-energy CT scanners to provide a virtual native image before contrast agent injection^22^. The image in 1 F looks exactly like the conventional attenuation image but the regions where iodine has been identified are substituted by the value of the surroundings (which is the gray value of water in our case and could be the Hounsfield unit of non enhanced blood vessels at a clinical scanner). The underlying iodine map has been analyzed with a separate measurement of different iodine concentrations and the accuracy for four different concentrations between 1–5.3 mg/ml NaI was better than 0.7 mg/ml for decomposition into water and sodium iodide.

### Virtual monoenergetic images

With the parametrization given in Eq. 2 and the knowledge of the effective photon energy, the effective atomic number and the electron density, the attenuation coefficients can be calculated for any energy with Eq. 6. This has been done exemplary for virtual energies of ${E}_{1,2,3}^{{\rm{VMI}}}=25,\,70,\,120\,{\rm{keV}}$. For 25 keV the image looks very similar to the original measurement with an effective energy *E*_eff_ = 24.6 keV. The determined attenuation coefficients agree very well with the calculated theory values for 25 keV with an error smaller than 1.3%.

Also for the virtual energy of 70 keV, deviations from the theoretical values are smaller than 0.8% and the contrast between the different materials has decreased as expected for higher photon energies. Please note that the applied model does not consider K-edge discontinuities and thus no increase in iodine contrast can be expected in the virtual monoenergetic images above the K-edge of iodine.

At 120 keV the virtual monoenergetic image looks very similar to the phase-contrast image and matches the literature values with an error less than 0.6%. While the photo effect is the dominating interaction mechanism at low photon energies, the Compton effect contribution is increasing towards higher energies. Just like the phase image, the Compton effect is proportional to the electron density resulting in the similar appearance of these two image signals.

### Demonstration with a chicken heart

**Figure 2:**
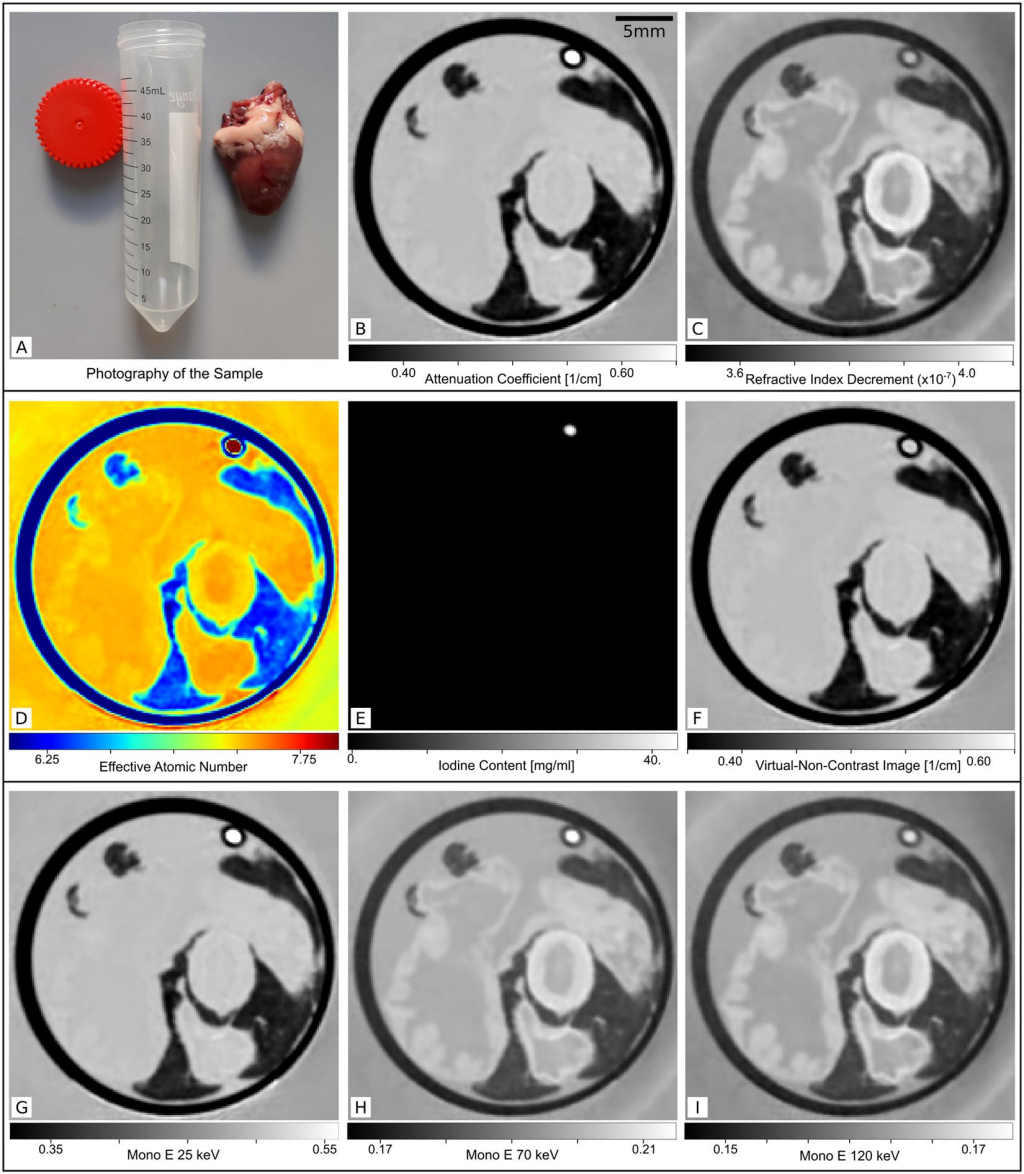
Measurements and results of virtual dual-energy processing for a biological soft tissue sample (chicken heart). (**A**) Photography of a fresh chicken heart next to the measurement container. The conventional attenuation image (**B**) shows very limited contrast only between the fatty tissue and the experimentally simulated iodine filled vessel. The phase-contrast image (**C**) reveals good contrast for the different anatomical structures like muscle, fat and blood vessels (most likely the aorta and two vessels of the low pressure system) but no contrast between contrast agent and the surrounding water. The effective atomic number map (**D**) reflects the situation of the conventional attenuation image with very low variations between the different structures besides fat and contrast agent. The quantitative iodine map (**E**) shows positive iodine concentrations of [I] ≈ 43 mg/ml only for the region of the contrast agent filled tube. The virtual non-contrast image (**F**) is the conventional attenuation image with the iodine containing pixels replaced with the attenuation value of water. At ${{\rm{E}}}_{{\rm{1}}}^{{\rm{VMI}}}=25\,{\rm{keV}}$, the virtual monoenergetic image (**G**) looks very similar to the conventional attenuation image. For the low atomic number soft tissue materials the Compton effect dominates the image formation already at ${{\rm{E}}}_{{\rm{2}}}^{{\rm{VMI}}}=70\,{\rm{keV}}$ (**H**) and a difference to the virtual monoenergetic image at ${{\rm{E}}}_{{\rm{3}}}^{{\rm{VMI}}}=120\,{\rm{keV}}$ (**I**) and the phase-contrast image is only visible for the iodine filled tube.

The grating-based phase-contrast CT of a chicken heart demonstrates gain on image information in the case of soft-tissue X-ray imaging. In Fig. 2, the conventional attenuation image shows only some image contrast between the very low attenuation coefficients of fat and the plastic tube which appear dark in the image. All other tissues and water appear with a very similar gray value and can not be further distinguished. The small tube filled with contrast agent has the highest attenuation coefficient and appears white in the chosen gray value range. The phase-contrast image shows detailed contrast for different anatomic structures of the heart tissue due to the different electron densities of muscle, fat and the surrounding water. The effective atomic number map shows an increased value for the iodine contrast agent and quite similar effective atomic numbers for all other materials in accordance with the result of the attenuation image. In the iodine map, only the contrast agent filled tube appears with a positive value such that the virtual non-contrast image is just the conventional attenuation image with those pixels replaced by the attenuation coefficients of water. For the virtual monoenergetic images it is apparent that the Compton effect dominates the image formation process already at the intermediate energy of ${E}_{{\rm{2}}}^{{\rm{VMI}}}=70\,{\rm{keV}}$ for the low atomic number materials of this sample, such that the image looks very similar to the electron-density image (=refractive index decrement image).

## Discussion

The approach of grating-based phase-contrast CT provides simultaneous access to precise quantitative values for the linear attenuation coefficient and the refractive index decrement or the electron density with less than 1% deviation from literature values. The different material dependencies of the underlying photon-matter interactions lead to complementary information in the two images. This is demonstrated by the different visual impression as well as the comparison of measured material quantities. Based on this complementary information, the well-known dual-energy material decomposition approaches have been applied to grating-based phase-contrast imaging. With the applied interaction parametrization^1^, the effective atomic numbers have been calculated from the original attenuation and electron density data in good agreement with two different literature sources. Starting from the photo effect and Compton effect contributions as the reference coordinate system, the transformation into material specific coordinate systems has been demonstrated. By that the standard dual-energy image representations of iodine content images, virtual non-contrast and virtual mono energy images can be calculated, as shown in this study.

The quantitative calculation of the iodine content by material decomposition is with an uncertainty of 0.7 mg/ml slightly less accurate than reported for commercially available dual-energy scanners (0.2 mg/ml in^23^, 0.5 mg/ml in^24^). This can mostly be attributed to the rather simple model for the photon interactions. The applied model for the photon interactions has two major drawbacks. First, the parametrization of photo and Compton effect under the assumption of the separability of material and energy dependencies is an empiric approximation optimized for a range of specific materials and energies that does not describe the actual combined dependency of the X-ray attenuation on both material and energy on a physically validated basis. Additionally, the model neglects the contribution of the coherent scattering which accounts for about 10% of the total attenuation coefficient of water at 25 keV^25,26^. Thus, the model must be further optimized to reach the current state of the art of quantitative material decomposition. Further, the effective interaction energy determined from the PMMA rod is well suited for materials with similar attenuation properties but fails for materials with significantly deviating atomic composition and electron configuration. Especially for iodine which has a K-edge discontinuity at 33.17 keV, this deviation should be accounted for by prior calibration measurements or an additional energy calibration with a well-defined iodine sample.

However, these limitations do not arise from the grating-based phase-contrast imaging approach, but from the straightforwardness of the applied model. On the contrary, the method brings some specific advantages for image based material decomposition. Independent of the detector type, the images are perfectly registered due to the simultaneous acquisition. In contrast to dual-energy imaging, grating-based phase-contrast imaging provides the total attenuation coefficient at one energy spectrum and simultaneously the refractive index decrement. With the latter being proportional to the electron density, this important material quantity can directly be extracted from the measurement without further image processing and the errors this may introduce. In clinical X-ray imaging many questions are related to the differentiation of materials with different atomic numbers (e.g. contrast agents, calcifications, kidney stones) or tissues with different electron density (e.g. brain parenchyma, breast tissue, coagulated blood). Thus, the simultaneous and direct access to the linear attenuation coefficient, which varies strongly for materials with high atomic numbers, as well as the electron density, which can – so far – not directly be extracted from conventional CT, would be a great benefit in a diagnostic context. Especially in case of stroke diagnosis, the differentiation of the extravasation of contrast agent via the damaged blood brain barrier into the brain tissue after an interventional treatment versus a remaining blood clot is difficult in conventional CT^27^. While the Hounsfield numbers of the relevant iodine concentrations and coagulated blood can be very similar^28^, the strongly different electron density enables a clear differentiation in phase-contrast CT.

A further advantage in comparison to standard dual-energy techniques is the existence of the conventional attenuation image from the full energy spectrum to which the radiologists are trained.

Altogether, the method provides improved soft-tissue contrast via the phase-shift, structural information via the small angle scattering signal (which was not described in this study), as well as the conventional attenuation image. With the additional extraction of the quantitative material decomposition information - as clinically provided by dual-energy scanners - this sums up to a significant gain of information density in a single CT acquisition.

In this study the general feasibility of virtual dual-energy imaging based on phase-contrast CT data and the translation to a biomedical application has been demonstrated. The quasi monochromatic X-ray source with an X-ray energy below the absorption K-edge of iodine has been chosen to enable a quantitative verification of the presented results. For clinical imaging, the performance of the method at higher energies and for polychromatic spectra of conventional X-ray sources will have to be further investigated as a next step. The feasibility of the accurate determination of the effective atomic number and the electron density with polychromatic grating-based phase-contrast CT has previously been demonstrated^16,29^. The limiting parameter for the decomposition approach is the complementarity of the two signals which scales with the contribution of the photo-electric effect (see equation 4). Especially in the case of clinical material decomposition, where higher atomic number materials like calcium or iodine are of interest, the crucial complementarity of the attenuation and phase-contrast information are expected at relevant X-ray energies. Further, the noise behavior in comparison to state of the art dual-energy CT should be analyzed in future studies.

The technical realization of a Talbot-Lau interferometer makes the interferometric technique accessible at conventional X-ray sources without specific requirements for the beam coherence which was not possible with earlier attempts of image segmentation from phase-contrast data^30,31^. Thus, its biggest advantage is the compatibility with commercial X-ray scanners as demonstrated in^32,33^. And with the latest technical improvements in grating fabrication, also translation to a large field of view at clinically relevant energies is possible^34^. We therefore believe that the clinical relevance of grating-based X-ray phase-contrast imaging with its simultaneous access to three complementary imaging modalities and the technical compatibility with existing clinical X-ray machines will profit from the additional access to the novel image visualization methods as known from dual-energy CT.

## Materials and Methods

### Sample preparation

For the purpose of a quantitative benchmark measurement, a specific material phantom has been designed. It consists of four different well-known solid (s) and fluid (f) materials which are namely ethanol (f), polymethylmethacrylat (PMMA) (s), polyoxymethylen (POM) (s) and nylon (type 6) (s). Additionally, a tube with coagulated blood (f) and a sodium iodide solution (f) with a concentration of [NaI] = 5.9 mg/ml (corresponds to a pure iodine concentration of [I] ≈ 5.0 mg/ml) in water have been inserted in the circular sample holder disc (see Fig. 1A). For the second measurement, a fresh chicken heart was put into a cylindric plastic container together with an intravenous injection line filled with contrast agent. The chicken heart was obtained from a local butchery. The used contrast agent was IMERON 300 (*Bracco Imaging Deutschland GmbH, Konstanz, Germany*) which was diluted in water to an approximate iodine concentration of 40 mg/ml. The tube containing the heart was filled up with water and both measurements were performed with the samples immersed in a rectangular water container. The surrounding water prevents artifacts due to big changes in the electron density and thereby phase-shifts larger than 2*π*, known as phase wrapping. The water container stays in place for the reference measurements and guarantees the same spectral contributions as in the sample scan. However, this only of minor importance in the case of the quasi monochromatic source used here.

### Munich Compact Light Source

All measurements were performed at the Munich Compact Light Source (MuCLS) which is a recent type of a brillant laboratory X-ray source. It is based on the combination of a small electron storage ring and a resonantly driven high-finesse laser cavity (*λ*_Laser_ = 1064 nm). X-ray photons are produced by inverse Compton-scattering at a defined collision point of laser photons and electrons with a repetition rate of about 65 MHz. The X-ray energy can be selectively changed from 15–35 keV by tuning the electron energy from 25–45 MeV. The average photon flux during the tomographic measurements was 1.4⋅10^10^ photons/s and the X-ray energy was tuned to 25 keV with an intrinsic energy bandwidth of Δ*E*/*E* ≈ 3%. A more detailed description of the working principle and the performance characteristics of the Munich Compact Light Source can be found in^35^. The purpose of using this compact source with synchrotron-like beam properties was the validation of the performance of the presented quantitative material decomposition approach. The spectrum at the position of the sample was measured with an energy-dispersive Amptek X-123 detector (*Amptek Inc., Bedford, Massachusetts*) with an 500 m Si sensor and from that a mean energy of *E*_mean_ = 24.3 keV was calculated. The uncertainty of the calibration energies arises from the uncertainty of the measurement of the density of the PMMA calibration rod (*ρ*_PMMA_ = 1.189 ± 0.005 *g*/*cm*^3^).

### Grating Interferometer

The grating interferometer is situated at a distance of about 15 m from the X-ray source point (size: 45 × 45 m^2^ r.m.s., divergence angle: 4 mrad). The elliptic field-of-view is 62 × 74 mm. The Talbot interferometer is realized with a phase grating (G1) with a period of 4.92 m, duty cycle of 0.5 and a nickel filling height of 4.39 m providing a phase shift of *π*/2 for the design energy of 25 keV. Period, duty cycle and gold filling for the absorption grating are p_2_ = 5 m, 0.5 and 70 m. The inter-grating distance for the first fractional Talbot order is d = 248 mm. All tomographic images were acquired with a single photon counting Pilatus-200 K detector (*DECTRIS ltd., Baden, Switzerland*) with a 1 mm thick silicon sensor and an effective pixel size of *p*_eff_ = 160 m. The quantum efficiency at 25 keV for 1 mm Silicon is 42.4%. The phase-shift and attenuation data was acquired by moving the absorption grating in 7 discrete steps over one grating period and comparison of the resulting sinusoidal stepping curve with a reference curve without sample. A detailed description of the method can be found in^13^. The exposure time per step was 3s for both measurements. To fulfill the angular sampling requirements, depending on the sample size 350–380 projections were taken over 360°, resulting in a total measurement time of 4–6 h.

### Image processing

From the raw projection phase-stepping images, attenuation, dark field and differential phase-contrast projections were extracted with an expectation maximization algorithm as reported in^36^. The reconstruction was done independently by filtered backprojection with a Ram-Lak filter for the attenuation data and a Hilbert filter for the phase data^15^. All of the reported image analysis, namely the material decomposition, the calculation of the effective atomic number and the calculation of virtual monochromatic images was performed on the reconstructed attenuation and phase-contrast volume data sets. The dark-field information was not used in this study.

For the cylindrical material phantom a mean of 50 slices has been taken to improve the photon statistics. In the case of the chicken heart measurement, the raw images (attenuation image and phase-contrast image) have been post-processed with a dictionary-based denoising algorithm as proposed in^37,38^ to achieve a similar noise level as in the cylindrical phantom.

The described image processing chain yields two reconstructed data sets which are the three dimensional attenuation coefficient and refractive index decrement maps.

### Parametrization of photon interactions

The measured and reconstructed quantities *μ* and *δ* can be related to the electron density and an effective atomic number as described in the following. The phase shift introduced by a material is related to the refractive index decrement

1 $$\delta =\frac{{r}_{0}{h}^{2}{c}^{2}}{2\pi {E}^{2}}\,\sum \,{N}_{i}\,{f}_{i}^{0}(0)=\frac{{r}_{0}{h}^{2}{c}^{2}}{2\pi {E}^{2}}{\rho }_{{\rm{e}}}$$

with the classical electron radius *r*_0_, the Planck constant *h*, the speed of light *c* and *N*_*i*_ the atomic density of the element with index *i*. The right side of equation 1 holds true above absorption edges, where the electrons of an atom can be considered to be quasi-free and the real part of the atomic scattering factor ${f}_{i}^{0}(0)$ in forward direction can be replaced by the atomic number *Z*_*i*_ and thus $\sum \,{N}_{i}\,{f}_{i}^{0}(0)={\rho }_{{\rm{e}}}$^39^. By that phase-contrast tomography provides direct access to the spacial distribution of the electron density of the whole specimen.

Simultaneously, the stepping procedure extracts the total attenuation coefficient as known from conventional X-ray imaging. For the diagnostic energy range, it has been proposed in^1^ to model the total attenuation *μ*(*E*) by two basic photon interactions, namely Compton and photo effect. The parametrization of energy and material dependence can be expressed by

2 $$\mu (E)={a}_{{\rm{c}}}\,{f}_{{\rm{c}}}(E)+{a}_{{\rm{p}}}\,{f}_{{\rm{p}}}(E),$$

where *f*_p,c_ are the energy and *a*_p,c_ the material dependencies of Compton and photo effect, respectively. Klein and Nishina^40^ proposed a parametrization for the scattering of photons at quasi-free electrons such that

3 $${a}_{{\rm{c}}}\,{f}_{{\rm{c}}}(E)={\rho }_{{\rm{e}}}{\sigma }_{{\rm{KN}}}(E),$$

with the so called Klein-Nishina coefficient *σ*_KN_(*E*) which is tabulated for the relevant energies. The photo effect has in this approach been parametrized by

4 $${a}_{{\rm{p}}}\,{f}_{{\rm{p}}}(E)={\rho }_{{\rm{e}}}{C}_{{\rm{P}}}\frac{{Z}^{{C}_{{\rm{Z}}}}}{{E}^{{C}_{{\rm{E}}}}}\,\mathrm{.}$$

The empirical parameters *C*_P_, *C*_Z_ and *C*_E_ were adapted from^1^ for the used X-ray energy in a prior parameter fit such that *C*_P_ = 13.03⋅10^−24^, *C*_Z_ = 3.42 and *C*_E_ = 2.97. By that we have a model which relates the measured attenuation coefficient and the electron with the atomic number

5 $${Z}_{{\rm{eff}}}=\sqrt[{C}_{{\rm{Z}}}]{(\frac{\mu }{{\rho }_{{\rm{e}}}}-{\sigma }_{{\rm{KN}}})\cdot \frac{1}{{C}_{{\rm{P}}}}\cdot {E}^{{C}_{{\rm{E}}}}}.$$

By that the measured and reconstructed quantities *μ* and *δ* can be expressed as the atomic number or the effective atomic number for mixtures and composites alongside with the electron density. Thus, the interaction parametrization model in Eq. 2 provides access to an energy independent description. Inversely, the attenuation coefficients for any arbitrary energy can be calculated

6 $$\mu (E)={\rho }_{{\rm{e}}}{\sigma }_{{\rm{KN}}}(E)+{\rho }_{{\rm{e}}}{C}_{{\rm{P}}}\frac{{Z}^{{C}_{{\rm{Z}}}}}{{E}^{{C}_{{\rm{E}}}}}.$$

which is known as virtual monoenergetic imaging in the clinical application of dual-energy CT.

### Volume-space based change of basis

If we interpret the two dimensional space of *Z*_*eff*_ and *ρ*_*e*_ as a vector space spanned by the effective atomic number and the electron density we can change to any new vector basis with an algebraic transformation matrix. By choosing two materials as a new image basis, one can express the old images in means of material contribution from the respective material. In the above demonstrated case, the images were decomposed into the contribution of iodine (*v*_i_) and a second basis material (*v*_x_). Here we used water or blood, depending on the relevant decomposition task. The effective atomic number and the electron density of 20 mg/mlNaI (${i}_{{{\rm{Z}}}_{{\rm{eff}}},{\rho }_{{\rm{e}}}}$) as the first basis material and a second basis material (${x}_{{{\rm{Z}}}_{{\rm{eff}}},{\rho }_{{\rm{e}}}}$) have been calculated with the aforementioned equations based on literature values^19,20^. It has been shown that an actually diluted salt as a basis material yields more precise decomposition results as the density of 20 mg/mlNaI represents the density of the clinically relevant iodine solutions more precisely than a mixture of solid NaI and water^41^.

The transformation matrix from the photo/Compton coordinate system to water/iodine or blood/iodine images is

7 $${{\bf{N}}}^{-1}=\frac{1}{{i}_{{{\rm{Z}}}_{{\rm{eff}}}}{x}_{{\rho }_{{\rm{e}}}}-{x}_{{{\rm{Z}}}_{{\rm{eff}}}}{i}_{{\rho }_{{\rm{e}}}}}(\begin{array}{cc}{x}_{{\rho }_{{\rm{e}}}} & -{x}_{{{\rm{Z}}}_{{\rm{eff}}}}\\ -{i}_{{\rho }_{{\rm{e}}}} & {i}_{{{\rm{Z}}}_{{\rm{eff}}}}\end{array}).$$

The resulting images are multiplied by the material’s mass density in [mg/ml] for quantitative information. For more detailed description of vector basis transformation we refer to common linear algebra textbooks.

### Virtual non-contrast image

The iodine has been identified via threshold segmentation in the quantitative “iodine-only” image (i.e. the pixels with positive iodine content) and the corresponding pixels have been replaced by a suitable background value - in this case water - with adapted standard deviation in the conventional image. This simple approach is applicable to a material combination as used in the presented samples. For application in clinical imaging the image segmentation can be supported by probability estimation algorithms and geometric considerations on the vector space as proposed in^41–43^ to extract a three material decomposition from only two measured data sets.

## Data Availability

The data that support the findings of this study are available from the corresponding author upon reasonable request.
