# Publisher Correction: Direct quantitative material decomposition employing grating-based X-ray phase-contrast CT

**DOI:** 10.1038/s41598-019-46258-w

**Published:** 2019-07-25

**Authors:** Eva Braig, Jessica Böhm, Martin Dierolf, Christoph Jud, Benedikt Günther, Korbinian Mechlem, Sebastian Allner, Thorsten Sellerer, Klaus Achterhold, Bernhard Gleich, Peter Noël, Daniela Pfeiffer, Ernst Rummeny, Julia Herzen, Franz Pfeiffer

**Affiliations:** 1 Chair of Biomedical Physics, Department of Physics and Munich School of BioEngineering, Technical University of Munich, 85748 Garching, Germany; 2 Department of Diagnostic and Interventional Radiology, Klinikum rechts der Isar, Technical University of Munich, 81675 München, Germany; 3 Max-Planck-Institute of Quantum Optics, Hans-Kopfermann-Straße 1, 85748 Garching, Germany

Correction to: *Scientific Reports* 10.1038/s41598-018-34809-6, published online 06 November 2018

This Article contains typesetting errors.

In the Materials and Methods section, under the subheading ‘Munich Compact Light Source’,

“The spectrum at the position of the sample was measured with an energy-dispersive Amptek X-123 detector (*Amptek Inc., Bedford, Massachusetts*) with an 500 m Si sensor and from that a mean energy of *E*_mean_ = 24.3 keV was calculated.”

should read:

“The spectrum at the position of the sample was measured with an energy-dispersive Amptek X-123 detector (*Amptek Inc., Bedford, Massachusetts*) with an 500 µm Si sensor and from that a mean energy of *E*_mean_ = 24.3 keV was calculated.”

Furthermore, in the Materials and Methods section, under the subheading ‘Grating Interferometer’,

“The grating interferometer is situated at a distance of about 15 m from the X-ray source point (size: 45 × 45 m^2^ r.m.s., divergence angle: 4 mrad).”

should read:

“The grating interferometer is situated at a distance of about 15 m from the X-ray source point (size: 45 × 45 µm^2^ r.m.s., divergence angle: 4 mrad).”

“The Talbot interferometer is realized with a phase grating (G1) with a period of 4.92 m, duty cycle of 0.5 and a nickel filling height of 4.39 m providing a phase shift of *π*/2 for the design energy of 25 keV. Period, duty cycle and gold filling for the absorption grating are p_2_ = 5 m, 0.5 and 70 m.”

should read:

“The Talbot interferometer is realized with a phase grating (G1) with a period of 4.92 µm, duty cycle of 0.5 and a nickel filling height of 4.39 µm providing a phase shift of *π*/2 for the design energy of 25 keV. Period, duty cycle and gold filling for the absorption grating are p_2_ = 5 µm, 0.5 and 70 µm.”

“All tomographic images were acquired with a single photon counting Pilatus-200 K detector (*DECTRIS ltd., Baden, Switzerland*) with a 1 mm thick silicon sensor and an effective pixel size of *p*_eff_ = 160 m.”

should read:

“All tomographic images were acquired with a single photon counting Pilatus-200 K detector (*DECTRIS ltd., Baden, Switzerland*) with a 1 mm thick silicon sensor and an effective pixel size of *p*_eff_ = 160 µm.”

